# Comparison of bioelectrical body and visceral fat indices and anthropometric measures in relation to type 2 diabetes by sex among Chinese adults, a cross-sectional study

**DOI:** 10.3389/fpubh.2023.1001397

**Published:** 2023-11-07

**Authors:** Jiangshan He, Binbin Zhang, Yaqi Fan, Yuxue Wang, Mianzhi Zhang, Chunjun Li, Li Zhang, Pei Guo, Minying Zhang

**Affiliations:** ^1^School of Medicine, Nankai University, Tianjin, China; ^2^Dongfang Hospital, Beijing University of Chinese Medicine, Beijing, China; ^3^Tianjin Academy of Traditional Chinese Medicine Affiliated Hospital, Tianjin, China; ^4^Tianjin Union Medical Center, Tianjin, China; ^5^Tianjin First Central Hospital, Tianjin, China

**Keywords:** body mass index, waist-hip ratio, percentage of body fat, visceral fat area, type 2 diabetes, optimal cutoff value

## Abstract

**Objectives:**

We aim to compare the efficacies of the bioelectrical indices (percentage of body fat, PBF; visceral fat area, VFA) with the conventional anthropometric measures (body mass index, BMI; waist-hip ratio, WHR) for predicting type 2 diabetes (T2D) risk by sex and to determine the sex-specific optimal adiposity indices to predict the T2D risk.

**Design:**

Cross-sectional design.

**Setting:**

Tianjin First Central Hospital and Tianjin Union Medical Center, Tianjin, China.

**Participants:**

A total of 9,332 adults (41.35% men) undergoing physical examination.

**Primary and secondary outcome measures:**

T2D was defined using the WHO’s criteria: fasting blood glucose (FBG) ≥7.0 mmol/L and/or previous diagnosis of T2D. Height, weight, waist, hip, PBF, VFA, and fasting plasma glucose were measured.

**Results:**

All studied adiposity indices were associated with T2D among both males and females, and the observed associations differed by sex. The standardized aORs of BMI, WHR, PBF and VFA for T2D were 1.60 (95% CI 1.42–1.81), 1.43 (95% CI 1.25–1.64), 1.42 (95% CI 1.23–1.62) and 1.53 (95% CI 1.35–1.75) for females, and 1.47 (95% CI 1.31–1.66), 1.40 (95% CI 1.25–1.58), 1.54 (95% CI 1.36–1.74) and 1.47 (95% CI 1.31–1.65) for males, respectively. The AUCs of VFA, WHR and BMI were 0.743, 0.742 and 0.717 in women, respectively, whereas none of the indices had AUC larger than 0.70 in men. The AUCs were not significantly different between VFA and WHR, while both demonstrate larger AUCs than BMI and PBF in females (all *p* < 0.05). The optimal cutoff values of VFA, WHR, and BMI for T2D in women were 103.55 cm^2^, 0.905, and 24.15 kg/m^2^, respectively.

**Conclusion:**

Although BMI, WHR, and PBF and VFA as measured by bioelectrical impedance analysis (BIA) were all positively associated with T2D, their efficacy for predicting the risk of T2D differed by sex. VFA, WHR and BMI could be used as biomarkers to predict T2D risk in women, however none of the study indicators demonstrated favorable efficacy of predicting T2D risk in men.

## 1. Introduction

Prevention and control of diabetes remains a global public health priority (1, 2). Type 2 diabetes (T2D) poses a two to three-fold higher risk of premature death in Asia, which has approximately 55% of the world’s diabetic population (3, 4).Obesity has been demonstrated as a modifiable risk factor for T2D (5, 6), and adiposity indicators are increasingly used for early detection of T2D risk. Although many adiposity indicators are available in clinical and epidemiological practice and research, few studies have been conducted on the comparison of the efficacy of multiple predictive adiposity indicators for T2D risk to identify the optimal predictive adiposity indicators.

Body mass index (BMI), a recognized measure of general obesity, recommended by the World Health Organization (WHO), has been shown to be effective for predicting T2D risk (7). However, BMI can only assess whether a person is overweight but cannot indicate either the percentage of body fat to the bodyweight or where the body fat is accumulated (8). Therefore, the accuracy of BMI as the all-encompassing measure of adiposity is debated (9). Waist-hip ratio (WHR) is one of the measures of central obesity and has been demonstrated to be more reliable to predict T2D risk than BMI (10), indicating where the body fat accumulates rather than the body fat mass is related to T2D. In recent decades, bioelectrical impedance analysis (BIA) is increasingly used to estimate body composition by analyzing the impedance obtained when a weak current flows through the body. Compared with computerized tomography and dual-energy X-ray absorptiometry methods, the BIA for estimating body composition is more affordable, non-invasive and easy to conduct, making it feasible to be applied in large populations. Percentage of body fat (PBF) and visceral fat area (VFA) are the most commonly used adiposity indicators measured by BIA and have been shown to be correlated with computerized tomography measurements and dual-energy X-ray absorption (11, 12). PBF, which is the percentage of body fat weight relative to total body weight, explains the body fat content. Studies have suggested that PBF, as measured by BIA, is more predictive for T2D risk than BMI (13, 14). Still, it cannot identify where the body fat is stored. VFA, which measures the fat accumulated around important internal organs, including the liver, intestine, and pancreas, could distinguish between visceral fat and subcutaneous abdominal fat better than WHR. Further evidence showed that visceral fat accumulation was related to abnormal glucose and lipid metabolism (15, 16). However, very few studies have compared VFA, as measured by BIA, with other indicators associated with T2D. The effectiveness of VFA remains to be studied. Although both BIA-measured adiposity indicators and conventional anthropometric indicators have been widely used to detect T2D risk, very few studies have compared the differences in the efficacy of these indicators in predicting T2D risk and there is a particular lack of data in large populations.

A substantial number of studies examining the relationship between adiposity index and the T2D risk have implicated the sex differences in the relationship detected (17–19). In a cohort of the aged Chinese adults, BMI, with the cutoff points as 25.78 kg/m^2^ for males and 24.86 kg/m^2^ for females, demonstrated to be the best predictor of T2D compared with waist circumference (WC), waist-to-height ratio (WHtR), and visceral adiposity index (VAI) (20). Sex differences in the association between VFA and T2D were also observed in Korean adults (17, 18). Among Chinese adults aged ≥50 years, the best indicator of the relationship between obesity and T2D was WHtR for men and BMI for women, respectively (21). In addition, WHtR exhibited better predictive power for the risk of T2D and the related cardio-metabolic risk among Iranian adults (10, 22). Cross-sectional evidence from Chinese adults suggests sex-specific predictive cutoff values for WHR, PBF, and VFA on T2D (19). In China, the cutoff point for BMI is recommended at 28 kg/cm^2^ for both men and women (23, 24). The WHO report suggests 0.85 and 1.00 as the cutoff value for WHR in women and men, respectively (25, 26). Visceral obesity was defined as VFA ≥ 100 cm^2^ for Asians (27). To date, no cutoff value has been proposed for PBF. Considering sex difference in the relationship between adiposity indicators and T2D risk helps to identify more reliable sex-specific optimal indicators.

Although there is growing evidence for the positive association between obesity and T2D (15), very few studies have compared bioelectrical indices with conventional anthropometric measures to T2D among Asians. To fill this knowledge gap, we conducted a cross-sectional study among a large sample of Chinese adults undergoing medical examination to compare the bioelectrical indices (PBF, VFA) with the most commonly used anthropometric measurements (BMI, WHR) in predicting the risk of T2D by sex. Furthermore, by conducting BIA measurement in a large population, our research could provide relevant data on the associations between adiposity indices measured using BIA method and T2D, thus to determine the sex-specific optimal obesity indicators to predict the risk of T2D.

## 2. Materials and methods

### 2.1. Study design and population

The present study used part of the baseline data of the Cohort Study on Natural Population in Beijing-Tianjin-Hebei Region, China, a National K&D Program from the Chinese Ministry of Science and Technology. A multi-stage stratified cluster sampling among attendees at physical examination centers was used in Beijing, Tianjin, and Hebei province, China. In the first stage, we recorded the physical examination centers in tertiary care hospitals with more than 200 organizations, institutions, or companies registered for medical examinations per year. We then randomly selected 2 to 3 medical examination centers by systematic sampling. In the second stage, we selected the registered organizations, institutions, and companies with stable employees and categorized them by occupation (white collar, pink collar, and blue collar) for each selected medical examination center, and then proportionally selected 100–200 registered organizations, institutions and companies for each occupation category. Then, individuals of those selected organizations, institutions, and companies aged 18 years or older and who voluntarily participated in the survey and signed the informed consent were included. Individuals who met the following criteria were recruited into our survey: 1) 18 years or older and 2) voluntarily participated in the survey and signed the informed consent. Individuals were excluded if they 1) had recent or disease-associated (e.g., from diabetes or cancer) weight loss, 2) had heart pacemakers, and 3) could not stand independently. The current research included participants at two selected medical examination centers in Tianjin between September 2018 and January 2020.

The research protocol was reviewed and approved by the ethical committees from Tianjin First Central Hospital (No. 2017N052KY) and Tianjin Union Medical Center (No. 2018C02). Written informed consent was obtained prior to the interview from each participant. The research procedures were carried out strictly following the Declaration of Helsinki. All methods were carried out in accordance with relevant guidelines and regulations.

### 2.2. Measurement of anthropometric variables

Medical professionals of the selected medical examination centers performed measurements of height, weight, WC, hip circumference (HC), PBF, and VFA. Height and weight were measured while participants were barefoot and wearing light indoor clothing in standing position using standard instruments (GL-310, Seoul, Korea). Then with a soft nonstretchable tape, WC was measured at the level of the umbilicus at the end of gentle expiration, and HC was measured at the widest part of the hip at the level of the greater trochanter with the tape positioned at a level parallel to the floor. WHR was calculated as the WC divided by HC. PBF and VFA were measured by the segmental multifrequency bioelectrical impedance analysis (S-MFBIA) method using the Inbody multifrequency impedance plethysmograph body composition analyzer (Inbody-770, Seoul, Korea). The participant stood barefoot on the instrument’s foot electrode in a fully vertical position with underwear, shared the weight evenly on both legs, held the hand electrode with both hands, and was asked to refrain from speaking during measurement. Measurements were completed after the reading was stable. Participants were instructed to fast for at least 12 h before blood sampling the following morning. Total cholesterol (TC), triglyceride (TG), high density lipoprotein cholesterol (HDL-C) and low density lipoprotein cholesterol (LDL-C) were measured (Hitachi, Inc., Tokyo, Japan). Participant’s systolic blood pressure (SBP) and diastolic blood pressure (DBP) were also measured in a sitting position for the right arm after resting for 10 min.

To control the measurement errors in BIA caused by fluid instability (28), we asked each participant if they had chronic kidney disease (CKD), and no participant reported having CKD. In addition, the estimated glomerular filtration rate was normal for each participant. Third, participants were instructed to fast for at least 12 h and no strenuous activity before measurement the following morning. Although measurement errors caused by fluid instability of the study subjects could not be eliminated entirely in BIA measurements, changes of body water caused by CKD, exercise, sweating, and drinking were excluded in the current study (29).

### 2.3. Measurement of covariates

An investigator-administered questionnaire interview was conducted face-to-face to collect information including demographic characteristics (age, sex, ethnicity, marital status, highest education, occupation), menopause status, self-reported personal and family history of T2D, smoking, alcohol drinking, the frequency and accumulated minutes of physical exercise [never (less than once a week), occasional (less than 30 min per day and 1–2 days per week) and regular (at least 30 min per day and 3 days per week)]. The investigators were medical undergraduates or graduate students trained and assessed qualified before participating in the survey.

### 2.4. T2D definition

In the medical examination center, overnight fasting venous blood was collected and measured for fasting glucose concentration (Toshiba, TBA-120FR, Japan). T2D cases were defined using WHO’s criteria: fasting blood glucose concentration (FBG) ≥7.0 mmol/L (14) and/or previously diagnosed medical history of T2D.

### 2.5. Statistical analysis

Statistical analyzes were performed using the Statistical Package for Social Sciences (SPSS) version 24.0 (SPSS Inc., Chicago, IL, United Kingdom) and MedCalc (MedCalc Software, Mariakerke, Belgium). The normally distributed continuous variables were expressed as mean ± standard deviation (SD) and compared using t-test, while variables that were not normally distributed were described using median and interquartile range and compared using the rank-sum test. The categorical data were described by rate and proportion and compared using Chi-square test. Logistic regression analysis was conducted to investigate the crude and adjusted Odds Ratios (ORs) of adiposity indices correlated with T2D by sex. The adjusted ORs were calculated adjusting for potential confounding variables, including age, marital status, ethnicity, education level, occupation, smoking, alcohol drinking, exercise, family history of T2D, research center, TC, TG, HLD-C, LDL-C, SBP and DBP (6, 21). The strength of the correlation was expressed as odds ratio (OR) and 95% confidence interval (CI). Because the raw adiposity indices were in different units, they were converted to z scores (original data subtracted the average and then divided by the SD) prior to the analysis. The Z score distribution has a mean of 0 and an SD of 1. The ORs derived using z scores therefore indicated T2D risk increased by per SD of the adiposity index. To address the effect of menopause on T2D, we additionally conducted stratified analysis by menopause in women.

Receiver operating characteristic (ROC) analysis was used to compare the predictive validity, and the area under the ROC curve (AUC) was also measured to examine the screening power of each obesity index and to describe the probability that an index would correctly identify subjects with T2D. AUC was assessed with 0.5 as no power and 1.0 as perfect power. The method suggested by DeLong et al. (30) was used to test whether the differences between AUC values were statistically significant. Two-tailed values of *p* < 0.05 were considered statistically significant.

### 2.6. Patient and public involvement

Participants were not involved in the design of this study, the specific aims or the research questions, nor were they involved in the recruitment and conduct of the study. Participants were not involved in interpreting study results or write-up of the manuscript. There are no plans to disseminate the research results to study participants.

## 3. Results

### 3.1. Baseline characteristics of the participants

**Figure 1:**
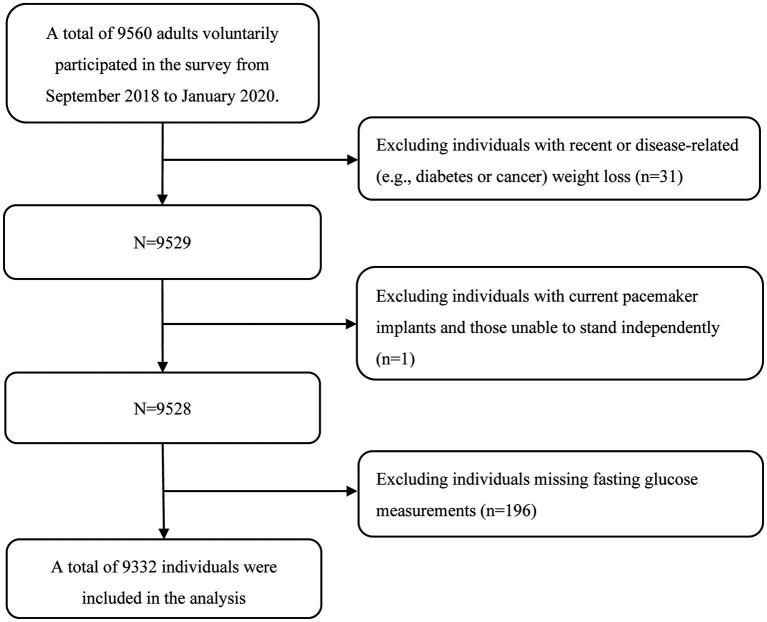
Flowchart of study population selection.

A total of 9,332 individuals (58.65% women) were included in the current analysis (Figure 1). The median age was 43.0 (IQR: 33.0, 56.0) years, ranging from 20 to 91 years. In the total study population, 91.89% had a college or higher degree, 38.33% were professionals, and 81.63% were in a current marriage.

The prevalence of T2D was 8.30%. The mean BMI, WHR, PBF, and VFA were 24.02 kg/m^2^, 0.89, 29.64%, and 92.49 cm^2^, respectively. Compared with women, men were characterized by a higher prevalence of T2D (11.66% vs. 5.94%), smoking (24.70% vs. 1.24%), alcohol drinking (33.25% vs. 4.31%), and having significantly higher VFA, BMI, WHR, SBP, DBP and TG, but lower PBF, HDL-C and LDL-C (*p* < 0.001). All demographic and clinical characteristics were stratified by sex and presented in Table 1. Comparison of the participants’ demographic and clinical characteristics by research center is presented in Supplementary Table S1.

**Table 1:** Baseline characteristics of the participants.

Characteristics	Total (9332)	Men (3859)	Women (5473)	*p*
Age (year), *n* (%)				<0.001
20–39	4,402 (47.17)	1747 (45.27)	2,655 (48.51)	
40–59	3,227 (34.58)	1,278 (33.12)	1949 (35.61)	
60–91	1703 (18.25)	834 (21.61)	869 (15.88)	
Educational level, *n* (%)				0.001
Middle school or below	757 (8.11)	360 (9.33)	397 (7.26)	
College or undergraduate	5,492 (58.85)	2,237 (57.97)	3,255 (59.47)	
Postgraduate or above	3,083 (33.04)	1,262 (32.70)	1821 (33.27)	
Han ethnicity, *n* (%)	8,989 (96.32)	3,736 (96.81)	5,253 (95.98)	0.035
Occupation, *n* (%)				<0.001
Civil servant	2,170 (23.25)	940 (24.36)	1,230 (22.47)	
Professionals	3,577 (38.33)	1,366 (35.40)	2,211 (40.40)	
Retired staff	1768 (18.95)	778 (20.16)	990 (18.09)	
Other occupation	1817 (19.47)	775 (20.08)	1,042 (19.04)	
Marital status, *n* (%)				<0.001
Unmarried	1,534 (16.44)	574 (14.88)	960 (17.54)	
Married	7,618 (81.63)	3,239 (83.93)	4,379 (80.01)	
Divorced/widowed	180 (1.93)	46 (1.19)	134 (2.45)	
Smoking, *n* (%)	1,021 (10.94)	953 (24.70)	68 (1.24)	<0.001
Drinking, *n* (%)	1,519 (16.28)	1,283 (33.25)	236 (4.31)	<0.001
Exercise, *n* (%)				<0.001
Regularly	2,578 (27.62)	1,362 (35.29)	1,216 (22.22)	
Sometimes	3,612 (38.71)	1,414 (36.64)	2,198 (40.16)	
No	3,142 (33.67)	1,083 (28.07)	2059 (37.62)	
Family history of diabetes, *n* (%)				0.010
Yes	2,173 (23.29)	838 (21.72)	1,335 (24.39)	
No	6,818 (73.06)	2,873 (74.45)	3,945 (72.08)	
Not quite clear	341 (3.65)	148 (3.83)	193 (3.53)	
Research center, *n* (%)				<0.001
Tianjin First Central Hospital	4,863 (52.11)	1862 (48.25)	3,001 (54.83)	
Tianjin Union Medical Center	4,469 (47.89)	1997 (51.75)	2,472 (45.17)	
Type 2 diabetes, *n* (%)	775 (8.30)	450 (11.66)	325 (5.94)	<0.001
Fasting plasma glucose, mmol/L	5.32 ± 1.10	5.45 ± 1.24	5.23 ± 0.98	<0.001
Obesity index				
PBF, %	29.64 ± 6.55	26.09 ± 5.78	32.14 ± 5.88	<0.001
VFA, cm^2^	92.49 ± 33.70	95.42 ± 32.31	90.42 ± 34.50	<0.001
BMI, kg/m^2^	24.02 ± 3.40	25.46 ± 3.15	23.01 ± 3.20	<0.001
WHR	0.89 ± 0.06	0.92 ± 0.06	0.88 ± 0.05	<0.001
Blood pressure, mmHg				
SBP	121.62 ± 17.77	126.88 ± 16.82	117.87 ± 17.47	<0.001
DBP	74.81 ± 10.99	78.36 ± 10.97	72.29 ± 10.28	<0.001
Blood lipid, mmol/L				
TC	4.85 ± 0.96	4.83 ± 0.93	4.86 ± 0.98	0.148
TG	1.38 ± 0.98	1.54 ± 1.15	1.26 ± 0.83	<0.001
HDL-C	1.41 ± 0.34	1.33 ± 0.29	1.47 ± 0.37	<0.001
LDL-C	2.88 ± 0.73	2.86 ± 0.72	2.90 ± 0.74	<0.001

PBF, percentage body fat; VFA, visceral fat area; BMI, body mass index; WHR, Waist-Hip Ratio; SBP, systolic blood pressure; DBP, diastolic blood pressure; TC, total cholesterol; TG, triglyceride; HDL-C, high density lipoprotein cholesterol; LDL-C, low density lipoprotein cholesterol.

### 3.2. Association of adiposity indices with risk of T2D by sex

Table 2 shows the standardized crude odds ratio (OR) and standardized OR adjusted for age, marital status, ethnicity, education level, occupation, research center, smoking, alcohol drinking, exercise, family history of T2D, research center, TC, TG, HLD-C, LDL-C, SBP and DBP by sex. Both in men and women, the crude and adjusted ORs of each adiposity index for T2D were significantly higher than the reference level of 1.00, suggesting a 1-SD increase of each index was associated with elevated odds of T2D (*p* < 0.001). As shown in the Supplementary Table S2, multivariate logistic regressions by research center and sex indicated that the study obesity indices are all associated with T2D among both men and women in the two research centers. Stratified analyzes by menopause demonstrated that the adjusted OR of each adiposity index for T2D remained statistically significant in both post-menopausal and the other women (Supplementary Table S3).

**Table 2:** Odds ratios (ORs) and 95% confidence interval (CI) of the adiposity indices for type 2 diabetes by sex.

		Men	Women
BMI	Crude OR	1.15 (1.12–1.19)^⁎^	1.27 (1.23–1.31)^⁎^
	Adjusted OR	1.13 (1.09–1.17)^⁎^	1.16 (1.12–1.20)^⁎^
BMI z-score	Crude OR	1.56 (1.42–1.72)^⁎^	2.12 (1.92–2.34)^⁎^
	Adjusted OR	1.47 (1.31–1.66)^⁎^	1.60 (1.42–1.81)^⁎^
WHR	Crude OR	1.13 (1.16–1.20)^⁎^	1.29 (1.18–1.33)^⁎^
	Adjusted OR	1.12 (1.05–1.20)^⁎^	1.18 (1.12–1.25)^⁎^
WHR z-score	Crude OR	1.80 (1.63–1.99)^⁎^	2.41 (2.15–2.69)^⁎^
	Adjusted OR	1.40 (1.25–1.58)^⁎^	1.43 (1.25–1.64)^⁎^
PBF	Crude OR	1.12 (1.10–1.14)^⁎^	1.14 (1.12–1.16)^⁎^
	Adjusted OR	1.08 (1.06–1.10)^⁎^	1.06 (1.04–1.09)^⁎^
PBF z-score	Crude OR	1.90 (1.71–2.11)^⁎^	2.16 (1.91–2.43)^⁎^
	Adjusted OR	1.54 (1.36–1.74)^⁎^	1.42 (1.23–1.62)^⁎^
**VFA**	Crude OR	1.02 (1.02–1.02)^⁎^	1.03 (1.02–1.03)^⁎^
	Adjusted OR	1.01 (1.01–1.02)^⁎^	1.01 (1.01–1.02)^⁎^
VFA z-score	Crude OR	1.90 (1.72–2.10)^⁎^	2.34 (2.11–2.60)^⁎^
	Adjusted OR	1.47 (1.31–1.65)^⁎^	1.53 (1.35–1.75)^⁎^

Symbols denote the significant of ORs (^⁎^*p* < 0.001).

BMI, body mass index; WHR, Waist-Hip Ratio; PBF, percentage body fat; VFA, visceral fat area.

ORs were adjusted for age, marital status, ethnicity, education level, occupation, research center, smoking, alcohol drinking, exercise, family history of diabetes, systolic and diastolic blood pressure, TC, TG, HDL-C and LDL-C.

PBF (1.54, 95%CI 1.36–1.74) demonstrated the largest adjusted odds ratio (aOR) for T2D in males, followed in descending order by VFA (1.47, 95%CI 1.31–1.65), BMI (1.47, 95%CI 1.31–1.66) and WHR (1.40, 95%CI 1.25–1.58), whereas BMI (1.60, 95%CI 1.42–1.81) demonstrated the largest aOR for T2D among females, followed in descending order by VFA (1.53, 95%CI 1.35–1.75), WHR (1.43, 95%CI 1.25–1.64) and PBF (1.42, 95%CI 1.23–1.62).

### 3.3. Roc analysis by sex

**Figure 2:**
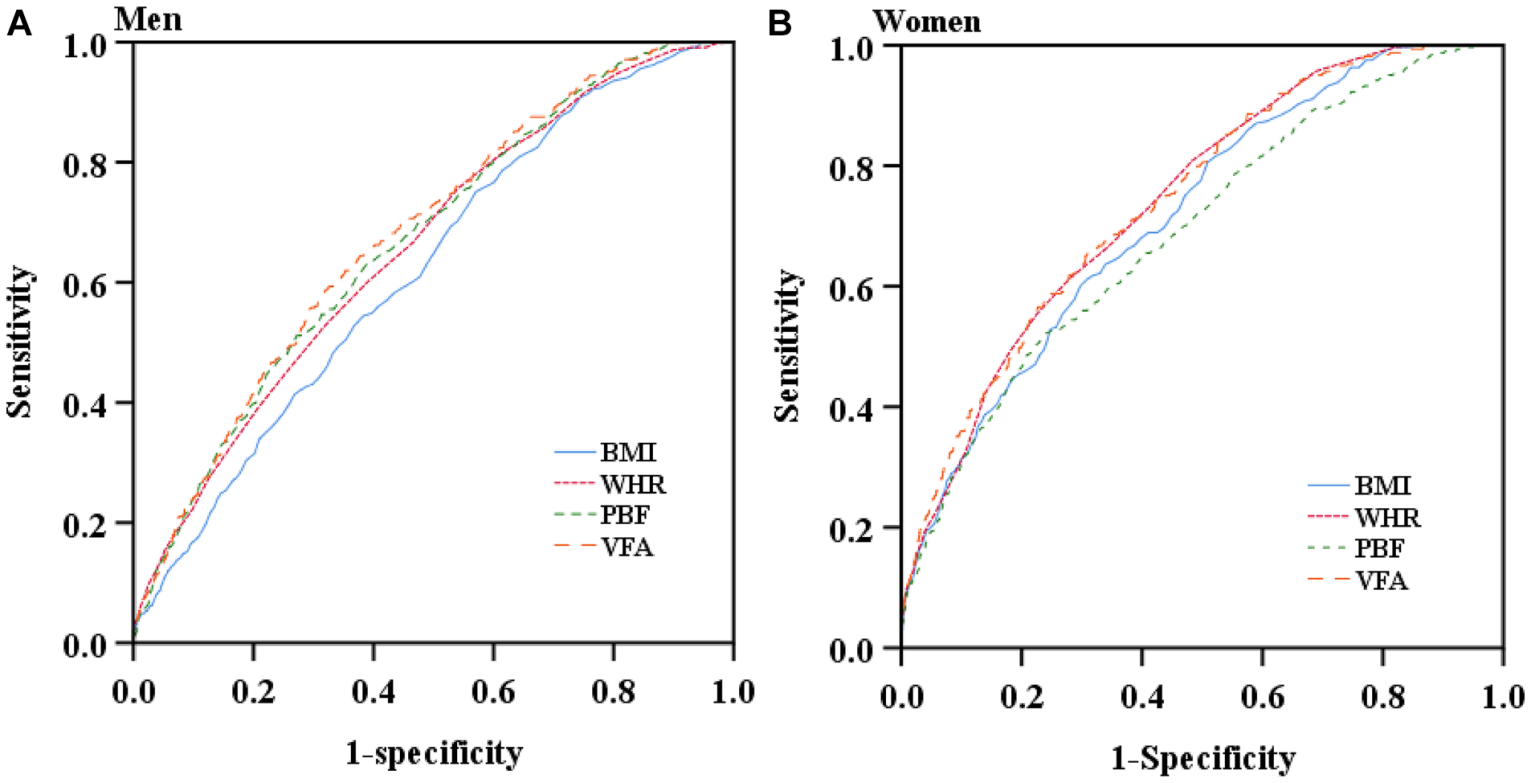
Receiver operating characteristic curves of the studied obesity indices for screening type 2 diabetes by sex. **(A)** Receiver operating characteristic curves of the studied obesity indices for screening type 2 diabetes in men. **(B)** Receiver operating characteristic curves of the studied obesity indices for screening type 2 diabetes in women.

Table 3 and Figure 2 are presented the AUCs, optimal cut-off values, sensitivities, specificities, and Youden indexes at the optimal cutoffs of the studied adiposity indices for identifying T2D by sex. All adiposity indices demonstrated significant identifying power for T2D (AUCs >0.5). VFA showed the largest AUCs among all studied adiposity indices in males and females (0.679 and 0.743, respectively). In addition, the AUCs of all studied indices were lower than 0.7 in men, whereas only the AUC of PBF was lower than 0.7 in women. The optimal cut-off value of VFA, WHR, and BMI in women were 103.55 cm^2^, 0.905, and 24.15 kg/m^2^, respectively. To address the impact of menopause, we additionally performed subgroup ROC analyzes by menopause among women. The results suggested that all indices were significant in identifying T2D (AUCs >0.5) and demonstrated higher AUCs among pre- and peri-menopausal women than post-menopausal women (Supplementary Table S4).

**Table 3:** Receiver operating characteristic curve analysis of the obesity indices for screening type 2 diabetes by sex.

	AUC (95% CI)	*p*	Optimal cutoff point	Sensitivity (%)	Specificity (%)	Youden index
*Men*						
BMI (kg/m^2^)	0.621 (0.595–0.647)	<0.001	24.65	75.11	42.95	0.1806
WHR	0.658 (0.632–0.683)	<0.001	0.905	75.56	46.05	0.2161
PBF (%)	0.668 (0.643–0.694)	<0.001	27.25	63.33	60.87	0.2420
VFA (cm^2^)	0.679 (0.654–0.704)	<0.001	105.25	59.11	67.67	0.2678
*Women*						
BMI (kg/m^2^)	0.717 (0.690–0.744)	<0.001	24.15	61.23	69.09	0.3033
WHR	0.742 (0.716–0.767)	<0.001	0.905	61.23	72.14	0.3338
PBF (%)	0.690 (0.660–0.719)	<0.001	35.95	52.31	75.84	0.2814
VFA (cm^2^)	0.743 (0.717–0.769)	<0.001	103.55	65.54	69.06	0.3459

BMI, body mass index; WHR, Waist-Hip Ratio; PBF, percentage body fat; VFA, visceral fat area.

### 3.4. Comparison of ROC curves for the studied adiposity indices in predicting the T2D risk by sex

As shown in Table 4, VFA was superior to BMI and WHR in men and BMI and PBF in women for predicting the risk of T2D (*p* < 0.05). The differences of ROC curves between VFA and PBF in men and VFA and WHR in women were not statistically significant, suggesting that there were no significant differences between VFA and PBF for identification of T2D in men and between VFA and WHR for identification of T2D in women.

**Table 4:** *p* values of pairwise comparisons of ROC curves for BMI, WHR, PBF and VFA in predicting the risk of type 2 diabetes by sex.

	Men				Women			
	BMI	WHR	PBF	VFA	BMI	WHR	PBF	VFA
BMI	1.000	0.002	<0.001	<0.001	1.000	0.038	0.006	0.002
WHR		1.000	0.305	0.011		1.000	<0.001	0.866
PBF			1.000	0.224			1.000	<0.001
VFA				1.000				1.000

## 4. Discussion

The current study revealed that although the studied adiposity indices were all positively associated with T2D, their screening efficacies for the risk of T2D were different and varied by sex. None of the indices studied could be sufficient to screen for T2D in men. However, VFA, WHR, and BMI could be potential indicators for predicting the risk of T2D among women, and VFA and WHR were superior to BMI.

BMI is the most widely used adiposity index that has been positively linked with the risk of T2D by a substantial number of cohort studies (31). Although research indicated that PBF might be a better predictor of T2D than BMI (14), very few studies have compared the screening efficacies of those available adiposity indices for T2D and no optimal indicator has been recommended. However, the current findings suggest that VFA, PBF and WHR may be better than BMI, and PBF may be inferior to VFA and WHR for screening T2D in both women and men. Excess visceral abdominal adiposity and subcutaneous abdominal adiposity are key contributors to abdominal obesity, which is recognized metabolic risk, but differ in structural composition, metabolic activity, and functional significance (32). Excess visceral adiposity is clearly associated with increased risks of metabolic syndrome. In contrast, there has been much debate regarding the role of subcutaneous abdominal adiposity (33, 34). BMI can only evaluate general obesity because it cannot differentiate between muscle and fat mass. Previous studies in China (19), Iran (10), Bangladesh (35) and Mexico (14) have also suggested the lower efficacy of BMI in identifying or predicting the T2D risk compared with other anthropometric or bioelectrical indices. The results of this study indicated that the efficacy of BMI to screen for T2D is inferior to WHR, PBF and VFA, as measured by BIA, in men and inferior to WHR and VFA in women. However, some studies reported contrasting results that BMI was the strongest predictor of T2D (20, 36). Although the results on which obesity index is the best for predicting the risk of T2D are inconsistent, the current study indicated the superiority of VFA, PBF, as measured by BIA, and WHR in males and VFA and WHR in females to BMI among Chinese adults. VFA measured by BIA demonstrated the largest AUCs for both men and women, 0.679 (0.654–0.704) for men and 0.743 (0.717–0.769) for women.

The present research revealed the sex differences in the screening efficacies of adiposity indices for T2D, even though there are inconsistent findings of sex differences in the associations between adiposity distribution and cardiometabolic disorders (37). A study among overweight or obese participants demonstrated that the male pattern of fat distribution is associated with higher cardiometabolic risk markers compared to women of similar age and BMI; however, visceral adipose tissue in women is more detrimental to cardiometabolic health, while lower extremity fat is comparatively more protective in women than in men (38). In contrast, a cross-sectional study implied that lower extremity fat is more protective of cardiometabolic risk in men than women. For both men and women, central obesity was more strongly associated with cardiovascular risk factors than other fat distributions (39). In the current study, although both the studied bioelectrical indices and the traditional anthropometric measures were associated with the risk of T2D in men and women, sex differences in the ability to predict the risk of T2D were observed for the obesity indices studied. We speculated that the sex differences might be related to the sexual structure of the human body and the different levels of sex hormones (40). Sex differences were previously observed in adiposity distribution and the prevalence and underlying mechanisms for related cardiovascular risk (37, 41, 42). The available evidence suggests that women had higher body fat mass than men (37), and insulin-stimulated glucose uptake is higher in women than men, suggesting that women generally show higher insulin sensitivity. This may contribute to a more healthy adipose tissue distribution, that is, more subcutaneous and less visceral fat in women than men (43). Indeed, research has shown that women store excess energy in a different way than men, with relatively more subcutaneous adipose tissue, mainly in the hips and thighs, while less adipose tissue is stored in visceral adipose tissue in the abdomen (44). The apple-shaped central fat distribution is more common in men and is associated with a higher risk of cardiometabolic disease, while pear-shaped peripheral fat distribution is more common in women and may prevent cardiometabolic disease (45). Sexual dimorphism in adipose distribution contributes considerably to sex differences in cardiometabolic health (44). In addition, estrogen having insulin-sensitizing (46) and anti-inflammatory (47) properties may also account for the sex difference in the associations between adiposity and cardiometabolic health outcome.

Sex differences of VFA for the risk of incident T2D were also identified in Korean adults (17). The VFA measure using BIA was correlated with a 72 and 325% increased T2D odds for men and women, respectively (18). The similar results were observed when VFA was measured by computed tomography (CT) scan, the ORs of VFA for T2D were 2.62 (95%CI: 1.73–3.97) and 32.49 (95%CI, 7.42–142.02) in men and women, respectively, and sex-specific cutoff points for VFA were proposed as 130 cm^2^ for men and 85 cm^2^ for women (17). Cross-sectional research in 3367 (2,307 male and 1,060 female) Chinese adults also observed sex differences in the ability of body composition (WHR, PBF, and VFA measured by BIA) to identify T2D (19). Inconsistent with our findings, the mentioned research did not find any AUC greater than 0.70 for WHR, PBF, or VFA, suggesting that none of these indicators was efficient in predicting the T2D risk.

In the current research, VFA demonstrated superiority with the largest AUCs among the studied indices in both men and women. Our findings were supported by a series of epidemiological and clinical studies, which showed that visceral fat, more than subcutaneous fat, correlates significantly and strongly with cardio-metabolic risks (48–50). For example, in Japan general population, absolute VFA value of about 100 cm^2^ was suggested to equate with obesity-related cardiovascular risk factor accumulation, irrespective of BMI (50). But visceral fat mass had been rarely used to assess obesity because it was not easy to measure. CT has been adopted as the gold standard for measuring visceral fat. However, CT is cost-intensive, complex, and involves radiation exposure, making it challenging to implement in large populations. Thus, it is urgent to develop a simple, non-invasive and more affordable method to assess visceral fat. In recent decades, BIA has been increasingly used because it is easy to conduct, non-invasive and much more affordable. Thus, PBF and VFA, as adiposity indices measured by BIA, are widely applied to predict the risk of cardio-metabolic abnormalities. Nevertheless, the consistency between BIA and CT is controversial. Although some research found VFA estimated by BIA is not fully consistent with CT measurements, and when using BIA to assess whether a person is visceral obesity, age, BMI and WC must be taken into consideration (51), a study demonstrated the positive correlation between VFA measured by BIA and CT, but the difference between VFA measured by CT and VFA measured by BIA increased with an advancing degree of obesity and a more accurate formula is needed to match CT data (11). It has also been shown that the segmental multifrequency bioelectrical impedance analysis (S-MFBIA) method, adopted by Inbody 770, has high concordance with Dual-Energy X-ray Absorptiometry when used to measure fat percentage, with the concordance correlation coefficient according to the four BMI groups (18.5–24.9, 25.0–29.9, 30–34.9, and ≥ 35.0 kg/m^2^) for PBF between 0.90–0.94 (12). Although the measurement of VFA using the BIA method has certain limitations, it has been extensively used in clinical, nutritional, sports practice and research (52–55) since the introduction of the S-MFBIA method. As such, by conducting S-MFBIA measurement in a large population, our research can provide relevant data on the associations between adiposity indices measured using S-MFBIA method and T2D.

To our best knowledge, the current study is the first to compare the associations between PBF, VFA, BMI, WHR, and T2D by sex in Asian adults. Furthermore, we determine the sex-specific optimal adiposity indices for predicting the T2D risk. In addition, a multi-stage stratified cluster sampling among attendees at physical examination centers was used to obtain a more diverse population on sociodemographic characteristics, lifestyle, obesity, and metabolic status, thus guaranteeing the representativeness of the sample and the high reliability of the findings. Third, our study sample’s age and gender profile are very close to that of the total Chinese population, which helps to determine the more reliable sex-specific index for T2D screening. However, this study also has some limitations. First, the current study only uses the baseline data of a cohort, thus preventing it from evaluating the temporal sequence and causal relationship between obesity and T2D. The findings need to be further verified by longitudinal data. Second, we assessed the participants’ PBF and VFA using the S-MFBIA method (Inbody 770). Studies have shown that the validity of body fat assessment by BIA might be influenced by sex, age, disease status, race and ethnicity, level of fatness, environment, phase of menstrual cycle, and underlying medical conditions (56, 57). Although we have used the validated BIA measurements for specific ethnic and racial groups, populations, measurement errors might not be completely control, especially in VFA measurement, suggesting that findings should be interpreted with caution when generalized to other races. Nevertheless, given the current extensive use of BIA methods in clinical, nutritional, sports practice and research (52–55), the current study may have practical significance by providing relevant data on adiposity indices measured using this method in relation to T2D risk. Third, this study has not adjusted for confounding factors, including dietary patterns and sex hormones, due to the lack of relevant information. Fourth, although FBG is simpler to define diabetes, it defines a few false-negative cases (58). Glucose tolerance and glycated hemoglobin tests were not performed in the current study. We thus fail to identify individuals with normal fasting glucose who would otherwise be diagnosed with T2D. Underestimations of both diabetes prevalence and the associations between the study adiposity indices and T2D might exist in this study. Fifth, a majority of the participants in this study had a high education level, which limited the generalization of the current findings. Caution should be taken when extrapolating the current results to populations with low educational levels. Despite the limitations, the findings have public health relevance and may be valuable in developing more accurate and specific public health recommendations and targeted preventative interventions.

## 5. Conclusion

In summary, we detected sex differences in the associations of the studied adiposity indices with T2D and the efficacy of predicting the T2D risk, although they were all associated with T2D in both men and women. VFA assessed by BIA, WHR, and BMI could be potential biomarkers for predicting T2D in women, and none of the studied indices showed sufficient efficacy for predicting T2D in men. The optimal cutoff values of VFA, WHR, and BMI for T2D in women were 103.55 cm^2^, 0.905, and 24.15 kg/m^2^, respectively.

## Data availability statement

The original contributions presented in the study are included in the article/Supplementary material, further inquiries can be directed to the corresponding author.

## Ethics statement

The studies involving humans were approved by the studies involving human participants were reviewed and approved by the ethical committees from Tianjin First Central Hospital (No. 2017N052KY) and Tianjin Union Medical Center (No. 2018C02). The studies were conducted in accordance with the local legislation and institutional requirements. The participants provided their written informed consent to participate in this study.

## Author contributions

MyZ has designed, supervised and oversaw the study implementation and takes responsibility for all aspects of the reliability and freedom from bias of the data presented and their discussed interpretation. MyZ and JH have written the manuscript. BZ conducted the analysis of the data. LZ, CL, and MzZ organized and managed the field work. JH, BZ, YF, YW, and PG participated in the investigation. All authors contributed to the article and approved the submitted version.
