# Supplementary material for: Comparison of bioelectrical body and visceral fat indices and anthropometric measures in relation to type 2 diabetes by sex among Chinese adults, a cross-sectional study
Source: Front Public Health. 2023 Nov 7;11:1001397. doi: 10.3389/fpubh.2023.1001397 (PMC10661931; doi:10.3389/fpubh.2023.1001397)
Supplement: Supplementary file 1 [file Table_1.DOCX]

Supplementary Material

# Supplementary Tables

**Table S1.** Baseline characteristics of the participants by research center.

| Characteristics | Total (9332) | Center A (4863) | Center B (4469) | *χ*^2^/*t* | *P* value |
| --- | --- | --- | --- | --- | --- |
| Sex |  |  |  | 39.289 | ＜0.001 |
| Men | 3859 (41.35) | 1862 (38.29) | 1997 (44.69) |  |  |
| Women | 5473 (58.65) | 3001 (61.71) | 2472 (55.31) |  |  |
| Age (year), n (%) |  |  |  | 139.085 | ＜0.001 |
| 20-39 | 4402 (47.17) | 2089 (42.96) | 2313 (51.76) |  |  |
| 40-59 | 3227 (34.58) | 1952 (40.14) | 1275 (28.53) |  |  |
| 60-91 | 1703 (18.25) | 822 (16.90) | 881 (19.71) |  |  |
| Educational level, n (%) |  |  |  | 751.295 | ＜0.001 |
| Middle school or below | 757 (8.11) | 407 (8.37) | 350 (7.83) |  |  |
| College or undergraduate | 5492 (58.85) | 3462 (71.19) | 2030 (45.42) |  |  |
| Postgraduate or above | 3083 (33.04) | 994 (20.44) | 2089 (46.74) |  |  |
| Han ethnicity, n (%) | 8989 (96.32) | 4721 (97.08) | 4268 (95.50) | 15.956 | ＜0.001 |
| Occupation, n (%) |  |  |  | 471.318 | ＜0.001 |
| Civil servant | 2170 (23.25) | 1065 (21.90) | 1105 (24.73) |  |  |
| Professionals | 3577 (38.33) | 1575 (32.39) | 2002 (44.80) |  |  |
| Retired staff | 1768 (18.95) | 870 (17.89) | 898 (20.09) |  |  |
| Other occupation | 1817 (19.47) | 1353 (27.82) | 464 (10.38) |  |  |
| Marital status, n (%) |  |  |  | 118.743 | ＜0.001 |
| Unmarried | 1534 (16.44) | 613 (12.61) | 921 (20.61) |  |  |
| Married | 7618 (81.63) | 4173 (85.81) | 3445 (77.09) |  |  |
| Divorced/widowed | 180 (1.93) | 77 (1.58) | 103 (2.30) |  |  |
| Smoking, n (%) | 1021 (10.94) | 575 (11.82) | 446 (9.98) | 8.128 | 0.004 |
| Drinking, n (%) | 1519 (16.28) | 620 (12.75) | 899 (20.12) | 92.747 | ＜0.001 |
| Exercise, n (%) |  |  |  | 333.128 | ＜0.001 |
| Regularly | 3142 (33.67) | 2052 (42.20) | 1090 (24.39) |  |  |
| Sometimes | 3612 (38.71) | 1671 (34.36) | 1941 (43.43) |  |  |
| No | 2578 (27.63) | 1140 (23.44) | 1438 (32.18) |  |  |
| Family history of diabetes, n (%) | |  |  | 57.156 | ＜0.001 |
| Yes | 2173 (23.29) | 1126 (23.15) | 1047 (23.43) |  |  |
| No | 6818 (73.06) | 3627 (74.58) | 3191 (71.40) |  |  |
| Not quite clear | 341 (3.65) | 110 (2.26) | 231 (5.17) |  |  |
| Type 2 diabetes, n (%) | 775 (8.30) | 342 (7.03) | 433 (9.69) | 21.578 | ＜0.001 |
| Obesity index |  |  |  |  |  |
| PBF, % | 29.64±6.55 | 29.19±6.52 | 30.13±6.56 | -6.925 | 0.006 |
| VFA, cm^2^ | 92.49±33.70 | 90.75±31.87 | 94.38±35.48 | -5.205 | ＜0.001 |
| BMI, kg/m^2^ | 24.02±3.40 | 23.93±3.21 | 24.12±3.60 | -2.734 | ＜0.001 |
| WHR | 0.89±0.06 | 0.89±0.06 | 0.90±0.06 | -4.263 | ＜0.001 |

BMI, body mass index; WHR, Waist-Hip Ratio; PBF, percentage body fat; VFA, visceral fat area.

**Table S2.** Standardized odds ratios (ORs) and 95% confidence interval (CI) for type 2 diabetes by research center and sex.

|  |  | Men | | Women | |
| --- | --- | --- | --- | --- | --- |
|  |  | OR | *P* | OR | *P* |
| Center A |  |  |  |  |  |
| BMI (kg/m^2^) | Crude OR | 1.39 (1.20-1.61) | ＜0.001 | 1.77 (1.54-2.04) | ＜0.001 |
|  | Adjusted OR | 1.30 (1.11-1.51) | 0.001 | 1.69 (1.46-1.96) | ＜0.001 |
| WHR | Crude OR | 1.57 (1.35-1.83) | ＜0.001 | 2.03 (1.74-2.37) | ＜0.001 |
|  | Adjusted OR | 1.40 (1.19-1.64) | ＜0.001 | 1.98 (1.67-2.34) | ＜0.001 |
| PBF (%) | Crude OR | 1.55 (1.33-1.81) | ＜0.001 | 1.64 (1.39-1.93) | ＜0.001 |
|  | Adjusted OR | 1.44 (1.22-1.70) | ＜0.001 | 1.57 (1.32-1.87) | ＜0.001 |
| VFA (cm^2^) | Crude OR | 1.88 (1.61-2.20) | ＜0.001 | 1.95 (1.69-2.26) | ＜0.001 |
|  | Adjusted OR | 1.73 (1.46-2.05) | ＜0.001 | 1.89 (1.62-2.22) | ＜0.001 |
| Center B |  |  |  |  |  |
| BMI (kg/m^2^) | Crude OR | 1.67 (1.47-1.90) | ＜0.001 | 2.54 (2.18-2.95) | ＜0.001 |
|  | Adjusted OR | 1.48 (1.28-1.72) | ＜0.001 | 2.03 (1.72-2.39) | ＜0.001 |
| WHR | Crude OR | 1.94 (1.69-2.22) | ＜0.001 | 2.93 (2.48-3.45) | ＜0.001 |
|  | Adjusted OR | 1.58 (1.36-1.85) | ＜0.001 | 2.22 (1.86-2.67) | ＜0.001 |
| PBF (%) | Crude OR | 2.16 (1.86-2.50) | ＜0.001 | 2.82 (2.36-3.38) | ＜0.001 |
|  | Adjusted OR | 1.87 (1.58-2.22) | ＜0.001 | 2.04 (1.68-2.47) | ＜0.001 |
| VFA (cm^2^) | Crude OR | 1.94 (1.71-2.20) | ＜0.001 | 2.80 (2.40-3.27) | ＜0.001 |
|  | Adjusted OR | 1.68 (1.45-1.95) | ＜0.001 | 2.15 (1.81-2.55) | ＜0.001 |

**Table S3.** Standardized odds ratios (ORs) and 95% confidence interval (CI) for type 2 diabetes among women by menopause.

|  |  | Pre- and primenopausal | Post-menopausal |
| --- | --- | --- | --- |
| BMI z-score | Crude OR | 1.98 (1.66-2.35) ^⁎^ | 1.71 (1.50-1.95) ^⁎^ |
|  | Adjusted OR | 1.87 (1.55-2.24) ^⁎^ | 1.63 (1.41-1.87) ^⁎^ |
| WHR z-score | Crude OR | 2.05 (1.67-2.50) ^⁎^ | 1.57 (1.37-1.81) ^⁎^ |
|  | Adjusted OR | 1.97 (1.58-2.44) ^⁎^ | 1.63 (1.41-1.88) ^⁎^ |
| PBF z-score | Crude OR | 1.67 (1.33-2.09) ^⁎^ | 1.58 (1.37-1.83) ^⁎^ |
|  | Adjusted OR | 1.68 (1.34-2.11) ^⁎^ | 1.45 (1.24-1.69) ^⁎^ |
| VFA z-score | Crude OR | 1.78 (1.49-2.14) ^⁎^ | 1.76 (1.54-2.01) ^⁎^ |
|  | Adjusted OR | 1.85 (1.52-2.25) ^⁎^ | 1.63 (1.42-1.88) ^⁎^ |

Symbols denote the significant of ORs (^⁎^p＜0.001).

BMI, body mass index; WHR, Waist-Hip Ratio; PBF, percentage body fat; VFA, visceral fat area.

ORs were adjusted for age, marital status, ethnicity, education level, occupation, research center, smoking, alcohol drinking, exercise, and family history of diabetes.

**Table S4.** Receiver operating characteristic curve analysis of the obesity indices for screening type 2 diabetes by menopause among women.

|  | AUC | *P* | Cut-off value | Sensitivity | Specificity | Youden index |
| --- | --- | --- | --- | --- | --- | --- |
| Non-menopausal women | |  |  |  |  |  |
| BMI (kg/m^2^) | 0.711 (0.659-0.762) | ＜0.001 | 21.85 | 81.48 | 48.14 | 0.2962 |
| WHR | 0.708 (0.657-0.759) | ＜0.001 | 0.875 | 65.43 | 63.16 | 0.2859 |
| PBF (%) | 0.637 (0.578-0.697) | ＜0.001 | 32.65 | 60.49 | 61.38 | 0.2188 |
| VFA (cm^2^) | 0.674 (0.621-0.728) | ＜0.001 | 69.55 | 86.42 | 42.95 | 0.2937 |
| Menopausal women | |  |  |  |  |  |
| BMI (kg/m^2^) | 0.627 (0.588-0.665) | ＜0.001 | 26.35 | 38.52 | 79.68 | 0.1820 |
| WHR | 0.605 (0.565-0.644) | ＜0.001 | 0.925 | 55.33 | 60.20 | 0.1552 |
| PBF (%) | 0.606 (0.566-0.647) | ＜0.001 | 36.55 | 55.33 | 63.34 | 0.1867 |
| VFA (cm^2^) | 0.636 (0.597-0.675) | ＜0.001 | 136.15 | 39.75 | 81.08 | 0.2083 |
